# Electrophysiological and pharmacological properties of GABAergic cells in the dorsal raphe nucleus

**DOI:** 10.1007/s12576-012-0250-7

**Published:** 2012-12-30

**Authors:** Yoshihiro Gocho, Atsushi Sakai, Yuchio Yanagawa, Hidenori Suzuki, Fumihito Saitow

**Affiliations:** 1 Department of Pharmacology, Nippon Medical School, Tokyo, 113-8602 Japan; 2 Department of Pediatrics, Nippon Medical School, Tokyo, 113-8602 Japan; 3 Department of Genetic and Behavioral Neuroscience, Gunma University Graduate School of Medicine and Japan Science and Technology Agency, CREST, Maebashi, 371-8511 Japan; 4 Japan Science and Technology Agency, CREST, Tokyo, 102-0075 Japan

**Keywords:** Dorsal raphe, GABAergic, Serotonin, Electrophysiology

## Abstract

The dorsal raphe nucleus (DRN) is the origin of the central serotonin [5-hydroxytryptamine (5-HT)] system and plays an important role in the regulation of many physiological functions such as sleep/arousal, food intake and mood. In order to understand the regulatory mechanisms of 5-HT system, characterization of the types of neurons is necessary. We performed electrophysiological recordings in acute slices of glutamate decarboxylase 67–green fluorescent protein knock-in mice. We utilized this mouse to identify visually GABAergic cells. Especially, we examined postsynaptic responses mediated by 5-HT receptors between GABAergic and serotonergic cells in the DRN. Various current responses were elicited by 5-HT and 5-HT_1A_ or 5-HT_2A/2C_ receptor agonists in GABAergic cells. These results suggested that multiple 5-HT receptor subtypes overlap on GABAergic cells, and their combination might control 5-HT cells. Understanding the postsynaptic 5-HT feedback mechanisms may help to elucidate the 5-HT neurotransmitter system and develop novel therapeutic approaches.

## Introduction

The dorsal raphe nucleus (DRN), which is the origin of the central serotonin (5-hydroxytryptamine; 5-HT) system, plays an important role in regulating many physiological functions, such as sleep/arousal, food intake, and mood. The DRN provides serotonergic innervation to several brain regions, including the cerebral cortex, basal forebrain, mesencephalon, hypothalamus, and thalamus, and it receives reciprocal inputs from these broad brain regions. The DRN is composed of heterogeneous neuronal groups that differ in cellular morphology, electrophysiological properties, and the expression of neurotransmitters, such as 5-HT, gamma-aminobutyric acid (GABA), and glutamate [1]. This heterogeneous organization may correspond to a variety of physiological functions that are involved in serotonergic neurotransmission and complicate the understanding of DRN functions. In addition to 5-HT cells, GABAergic cells consist of another major DRN cell group [1–3]. GABAergic cells function as interneurons in local circuits with serotonergic projection neurons and regulate their output [4]. Therefore, characterization of the properties of each cell type, especially of GABAergic cells, and their interactions may help to elucidate the various functions of the DRN.

To date, many investigations have described the properties of DRN 5-HT cells through in vivo or in vitro electrophysiological recordings with neurochemical identification [5–8]. However, these studies did not directly characterize the properties of DRN GABAergic cells but described GABAergic cells as putative non-5-HT cells that do not exhibit tryptophan hydroxylase (TPH) immunoreactivity. Since identifying GABAergic cells under electrophysiological recordings is difficult, the properties of DRN GABAergic cells have been poorly characterized.

In order to characterize GABAergic cells, we used a knock-in mouse line in which the expression of green fluorescent protein (GFP) was controlled by the glutamate decarboxylase (GAD) 67 endogenous promoter in order to specifically visualize GABAergic cells [9]. This transgenic mouse line (GAD67^+/GFP^) allows for the identification and characterization of GABAergic cells in the brain [10–12]. The electrophysiological and pharmacological properties of directly identified GABAergic cells in the DRN have not yet been characterized. Thus, this study aimed to characterize the differences in electrophysiological and pharmacological properties in GABAergic and non-GABAergic cells in the DRN of GAD67^+/GFP^ mice with whole-cell patch-clamp recording techniques and immunohistochemistry. The properties of GABAergic cells differed from those of non-GABAergic cells in the DRN. Furthermore, the DRN GABAergic cells were heterogeneous in their postsynaptic responses to 5-HT and selective agonists of 5-HT_1A_ and 5-HT_2A/2C_ receptors.

## Materials and methods

### Animals

The generation of GAD67–GFP knock-in mice have been described previously [9], and these mice, used in the present study, were termed GAD67^+/GFP^. The mice were maintained with a genetic background of C57BL/6 at our animal facility. In accordance with a protocol approved by the Ethics Review Committee of Nippon Medical School, we made efforts to minimize the number of animals used and their suffering.

### Immunohistochemistry

Male heterozygous GAD67^+/GFP^ mice aged 24 days (*n* = 2) were deeply anaesthetized with pentobarbital and transcardially perfused with freshly prepared 4 % paraformaldehyde in phosphate-buffered saline (PBS). The midbrain was removed, post-fixed at 4 °C overnight, and cryoprotected in 20 % sucrose in PBS at 4 °C overnight. The midbrain was cut into serial transverse cryosections with a cryostat (Leica, Tokyo, Japan). Immunohistochemistry was performed on free-floating sections, as previously described [13]. Briefly, transverse cryosections (20 μm) of the midbrain were incubated with a rabbit anti-GFP polyclonal antibody (1:2,000; EMD Millipore, MA, USA) and a sheep anti-TPH polyclonal antibody (1:2,000; Life Technologies, NY, USA) for 3 days at 4 °C. The sections were then incubated with secondary antibodies labeled with Alexa Fluor 594 (1:2,000; Life Technologies) for GFP and Alexa Fluor 488 (1:2,000; Life Technologies) for TPH. After electrophysiological recording, slices were immersion-fixed overnight in 4 % paraformaldehyde prepared in 0.1 M PBS and then stored in PBS. Fixed sections were incubated with a sheep anti-TPH polyclonal antibody (1:1,000; Life Technologies) for 3 days at 4 °C. Subsequently, these sections were incubated with an Alexa Fluor 488 (1:1,000)-conjugated donkey anti-sheep secondary antibody and streptavidin-conjugated Alexa Fluor 594 (1:1,000) to visualize the immunohistochemical labeling and biocytin. Images were captured with a high-resolution digital camera (Olympus, Tokyo, Japan).

### Electrophysiological recording

Slices for experiments were prepared from 21- to 27-day-old male GAD67^+/GFP^ mice. The mice were deeply anesthetized by halothane inhalation. Following decapitation, the brains were rapidly removed and placed in ice-cold Na^+^-deficient saline (~4 °C) that contained 252 mM sucrose, 21 mM NaHCO_3_, 3.35 mM KCl, 0.5 mM CaCl_2_, 6.0 mM MgCl_2_, 0.6 mM NaH_2_PO_4_, and 10 mM glucose. Coronal slices (250 μM) were cut with a vibratome (Leica) through the entire rostrocaudal extent of the DRN between −4.84 and −4.48 Bregma (according to the atlas of Paxinos and Franklin [26]) and placed in a submerged chamber for at least 1 h in artificial cerebrospinal fluid (ACSF) that contained 138.6 mM NaCl, 3.35 mM KCl, 2 mM CaCl_2_, 1.3 mM MgCl_2_, 21.0 mM NaHCO_3_, 0.6 mM NaH_2_PO_4_·2H_2_O, and 10.0 mM glucose. ACSF was maintained at pH 7.4 by bubbling 95 % O_2_/5 % CO_2_ gas.

Individual slices were transferred to a recording chamber attached to a microscope stage, continuously perfused with oxygenated ACSF at a flow rate of 1.5 mL/min, and maintained at room temperature (~27 °C). Borosilicate glass-patch electrodes (World Precision Instruments, FL, USA) with a resistance of 6–12 MΩ when filled with an internal solution of 150 mM potassium methanesulfonate, 1.0 mM KCl, 0.2 mM K-EGTA, 20 mM HEPES, 3.0 mM MgATP_2_, and 0.4 mM Na-GTP (pH 7.38) were used for whole-cell recordings of DRN cells. Cells that were visualized under a blue light were considered GFP-positive (GFP(+)), and these were designated GABAergic cells. Cells without GFP were considered GFP-negative (GFP(−)), and these were designated non-GABAergic cells. Whole-cell patch-clamp recordings were acquired and controlled with Axon 700B Multiclamp amplifier and pClamp acquisition software (Molecular Devices, CA, USA). In the pharmacological experiments, the amplitudes of membrane current induced by serotonergic agonists were measured from the baseline before application of agonist to peak current amplitude of their responses.

### Chemicals

The following chemicals were used in this study: 5-HT, (*R*)-(+)-8-hydroxy-2-(di-*n*-propylamino)tetralin hydrobromide (8-OH-DPAT), and (+/−)-2,5-dimethoxy-4-iodoamphetamine hydrochloride (DOI) (Sigma-Aldrich, MO, USA).

### Analysis and statistics

The data are presented as the mean ± standard error of the mean, and n represents the number of independent experiments. Statistical differences were evaluated with the Tukey–Kramer test. *P* values <0.05 were considered statistically significant.

## Results

### Distribution of GFP-positive cells and GFP-negative cells in the DRN of GAD67^+/GFP^ mice

**Fig. 1:**
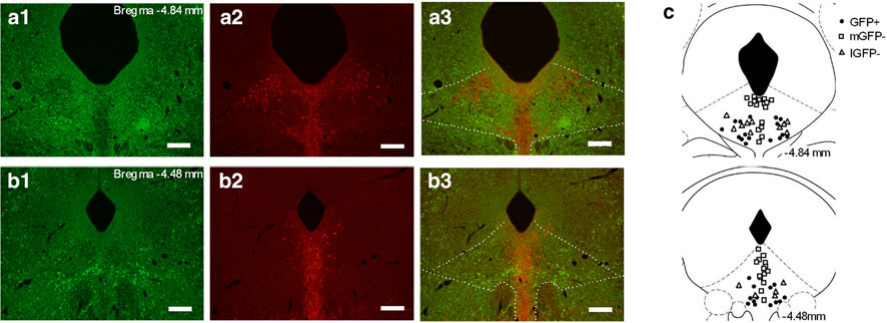
Distribution of GFP-positive neurons and TPH-containing neurons in the DRN. **a**, **b** Fluorescent images of GFP-positive neurons (*green*) and TPH-containing neurons (*red*) at the level of the caudal and rostral DRN. The *numbers in the upper right* of the images represent the distances from Bregma. GFP-positive neurons were present within lateral areas of the DRN and absent at the midline areas, whereas TPH-containing neurons were densely distributed at the midline areas. **c** Schematic of the sites of the neurons from which electrophysiological properties were obtained. The *different symbols* represent the types of tested neurons: GFP-positive neurons (GFP(+); *filled circle*), GFP-negative neurons located in medial DRN (mGFP(−); *square*), and GFP-negative neurons located in lateral DRN (lGFP(−); *triangle*). The *numbers on the lower right* of the coronal sections represent the distances from Bregma

**Fig. 2:**
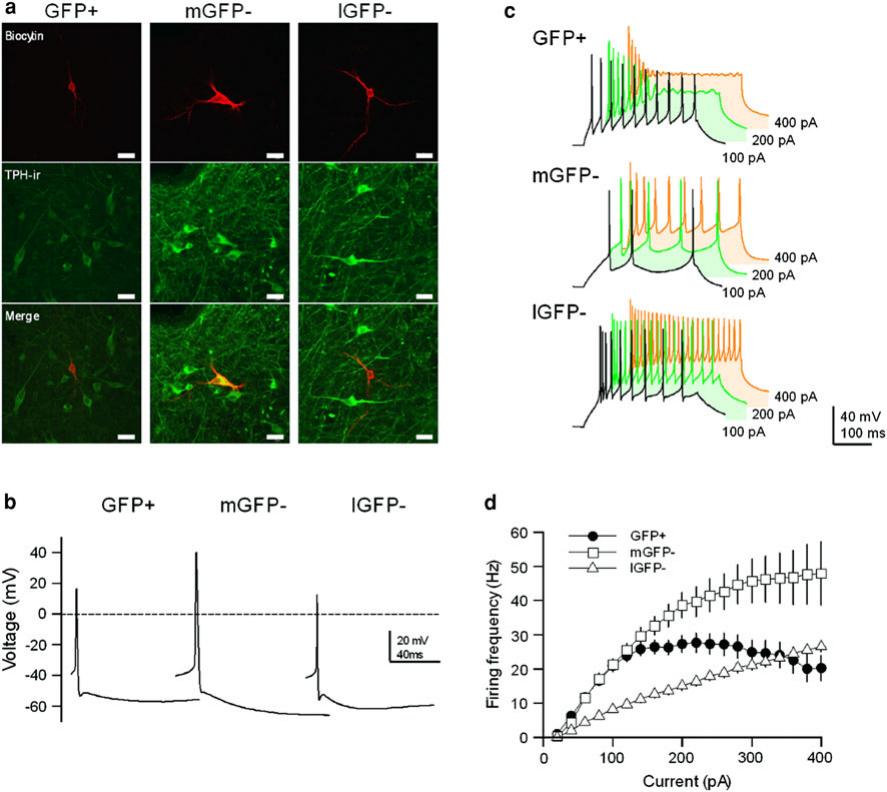
Morphological and electrophysiological differences among green fluorescent protein (GFP)-positive cells (GFP(+)), GFP-negative cells located in medial DRN (mGFP(−)), and GFP-negative cells located in lateral DRN (lGFP(−)). **a** Immunohistochemistry of the 3 types of DRN cells. The cell is double-labeled for both tryptophan hydroxylase (TPH; *green*) and biocytin (*red*). *Scale bar* 10 μm in both panels. **b** Representative action potential (AP) waveforms of GFP(+), mGFP(−), and lGFP(−) neurons. These APs were elicited by the minimum depolarizing holding current. **c** AP waveforms of GFP(+), mGFP(−), and lGFP(−) neurons in the DRN that were generated by injecting currents of 100 pA (*black*), 200 pA (*green*), and 400 pA (*orange*), respectively. **d** Input–output relationship curves of GFP(+), mGFP(−), and lGFP(−) neurons in the DRN (GFP(+), *n* = 39; mGFP(−), *n* = 35; lGFP(−), *n* = 18). APs were generated by injecting current steps from 0 to 400 pA in increments of 20 pA. The firing frequency of AP were calculated at the period of current injection (400 ms)

The DRN of GAD67^+/GFP^ mice contained a large population of brightly fluorescent GFP(+) cell structures, including the soma, dendrites, and axons (Fig. 1a1, b1). In GAD67^+/GFP^ mice, numerous GFP(+) cells were localized to the lateral DRN, whereas TPH-immunopositive cells were densely present within the midline DRN where GFP(+) cells were hardly observed. Numerous small (5–10 μm) GFP(−) cells that were TPH-immunonegative were intermingled with GFP(+) cells in the lateral DRN (Fig. 1a, b). This complementary distribution of GFP(+) cells and TPH-immunopositive cells was consistent with a previous study [3]. We obtained whole-cell patch-clamp recordings of the membrane properties of 74 GFP(+) cells and 73 GFP(−) cells in the DRN of GAD67^+/GFP^ mice. All tested GFP(+) cells were located in the lateral DRN. GFP(−) cells that were located in the midline DRN (mGFP(−)) accounted for two-thirds of the tested GFP(−) cells (37/55), and the remaining tested GFP(−) cells (18/55) were located in the lateral DRN (lGFP(−)) (Fig. 1c). After the recordings, 71 GFP(+), 45 mGFP(−), and 15 lGFP(−) biocytin-filled DRN cells were reconstituted and immunolabeled with TPH in order to determine whether the cell was a 5-HT cell (Fig. 2a). The majority of stained GFP(+) cells (77 %, 55/71) were TPH-immunonegative, whereas most stained mGFP(−) cells (91 %, 41/45) were TPH-immunopositive (5-HT cells). All stained lGFP(−) cells were TPH-immunonegative (putative glutamatergic cells, *n* = 15). However, 23 % of the GFP(+) cells (16/71) had weak TPH-immunoreactivity. These results were consistent with recent study that demonstrated the presence of GAD67-containing 5-HT neurons in the rat DRN [14], but this cell group is remained to be clarified. mGFP(−) cells had significantly larger cell bodies than those of GFP(+) cells and lGFP(−) cells. (soma cross-sectional area: mGFP(−), 162.0 ± 7.9 μm^2^; GFP(+), 87.9 ± 3.9 μm^2^; lGFP(−), 63.4 ± 5.0 μm^2^; mGFP(−) vs. GFP(+), *p* < 0.001; mGFP(−) vs. lGFP(−), *p* < 0.001; GFP(+) vs. lGFP(−), p > 0.05).

### Intrinsic membrane properties of DRN GFP(+) and GFP(−) cells

We analyzed the intrinsic membrane properties of 74 GFP(+), 55 mGFP(−), and 18 lGFP(−) cells in the DRN. Representative examples of action potential (AP) shapes of GFP(+), mGFP(−), and lGFP(−) cells are shown in Fig. 2b. Almost no DRN cells were spontaneously active. There were many significant differences in the multivariate analyses between them. The resting membrane potentials of GFP(+) and lGFP(−) cells were more depolarized than those of mGFP(−) cells (Table 1). The threshold potential for AP generation (AP threshold) of GFP(+) cells was significantly hyperpolarized (Table 1). In addition, input resistance was significantly larger in GFP(+) cells than in mGFP(−) and lGFP(−) cells (Table 1). The most distinguishable of these differences were the AP waveforms and amplitudes. As shown in Fig. 2b, mGFP(−) cells had larger AP amplitudes and wider AP waveforms compared to those of GFP(+) and lGFP(−) cells, whereas the AP waveforms of GFP(+) and lGFP(−) cells were similar. AP overshoot and amplitude of mGFP(−) cells were significantly larger than those of GFP(+) and lGFP(−) cells (Table 1). Regarding AP kinetics, such as half-width, rise time, and decay time, mGFP(−) cells had significantly broader APs compared to GFP(+) and lGFP(−) cells (Table 1). Although most cells had both fast and slow AHPs (fAHP and sAHP) components, some mGFP(−) cells were deficient in the fAHP. The sAHP amplitude of mGFP(−) cells was significantly larger than those of GFP(+) and lGFP(−) cells (Table 1). The fAHP amplitude of lGFP(−) cells was larger than those of GFP(+) and mGFP(−) cells (Table 1).

**Table 1:** Electrophysiological properties of GFP(+), medial GFP(−), and lateral GFP(−) cells in the DRN

	GFP+	m GFP−	l GFP−
Resting membrane potential (mV)	−69.8 ± 0.7 (74)	−76.6 ± 0.9*** (55)	−69.4 ± 1.3^∫∫∫^ (18)
Input resistance (MΩ)	756 ± 34^†^ (74)	638 ± 28* (55)	588 ± 54 (18)
AP threshold (mV)	−38.3 ± 0.5^†††^ (74)	−34.1 ± 0.5*** (55)	−31.8 ± 1.1 (18)
AP overshoot (mV)	14.1 ± 1.0 (74)	32.7 ± 0.7*** (55)	14.6 ± 1.8^∫∫∫^ (18)
AP amplitude (mV)	52.4 ± 1.0^††^ (74)	66.8 ± 0.7*** (55)	46.4 ± 1.6^∫∫∫^ (18)
fAHP amplitude (mV)	16.0 ± 0.7^†††^ (70)	17.0 ± 0.7 (37)	22.4 ± 0.9^∫∫∫^ (18)
sAHP amplitude (mV)	21.6 ± 0.5 (69)	29.4 ± 0.7*** (55)	24.2 ± 1.3^∫∫∫^ (18)
Half-width (ms)	0.70 ± 0.02 (74)	1.35 ± 0.04*** (55)	0.58 ± 0.04^∫∫∫^ (18)
Rise time 10–90 % (ms)	0.29 ± 0.01 (74)	0.44 ± 0.01*** (55)	0.25 ± 0.02^∫∫∫^ (18)
Decay time 90–10 % (ms)	0.42 ± 0.02 (74)	1.00 ± 0.04*** (55)	0.33 ± 0.03^∫∫∫^ (18)

Values indicate mean ± standard error of the mean. The numbers of data are indicated within parentheses. Special symbols (*, ^∫^, ^†^) indicate statistically significant differences between groups by the multiple-comparison test, the Tukey–Kramer test (*, ^∫^, ^†^ *p* < 0.05; **, ^∫∫^, ^††^ *p* < 0.01; ***, ^∫∫∫^, ^†††^ *p* < 0.001). *, ^∫^, and ^†^ correspond to GFP(+) different from mGFP(−), mGFP(−) different from lGFP(−), and lGFP(−) different from GFP(+), respectively

*GFP*(+) GFP positive, *GFP*(−) GFP negative, *mGFP*(−) GFP(−) cells in the medial DRN, *lGFP*(−) GFP(−) cells in the lateral DRN, *AP* action potential, *fAHP* fast after hyperpolarization, *sAHP* slow after hyperpolarization

Next, we injected positive current steps from 0 to 400 pA with increments of 20 pA (duration of 400 ms) to determine the active membrane properties of the cells (Fig. 2c). As shown in Fig. 2d, the input–output relationship curves of GFP(+) and lGFP(−) cells were steeper than that of mGFP(−) cells. GFP(+) and lGFP(−) cells had a similar sensitivity to injected currents <120 pA, but the firing frequency of GFP(+) cells saturated around 25 Hz with input currents over 140 pA. The firing frequency of APs that were generated by injecting 200 pA for 400 ms significantly differed among these 3 cell populations (GFP(+), 27.3 ± 2.5 Hz; mGFP(−), 15.1 ± 1.2 Hz; lGFP(−), 38.6 ± 3.7 Hz; GFP(+) vs. mGFP(−), *p* < 0.001; mGFP(−) vs. lGFP(−), *p* < 0.001; GFP(+) vs. lGFP(−), *p* < 0.01).

### Effects of 5-HT, 8-OH-DPAT, and DOI

**Fig. 3:**
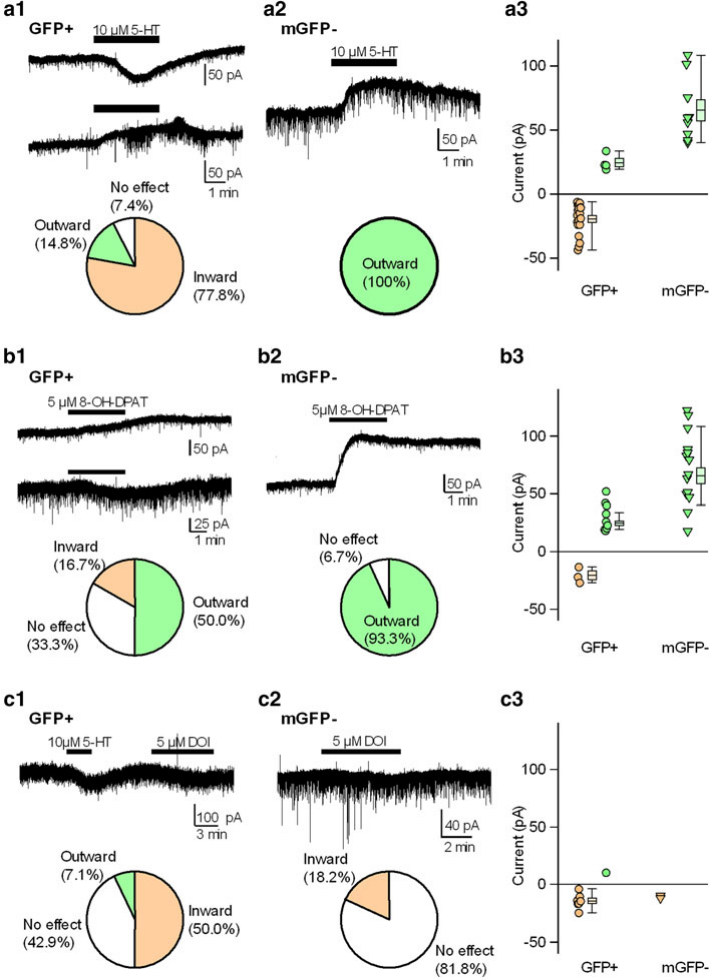
Effects of 5-HT, 8-OH-DPAT, and DOI on membrane currents of GFP-positive and medial GFP-negative cells in the DRN. Representative current traces of GFP(+) (**a1**, **b1**, **c1**) and mGFP(−) (**a2**, **b2**, **c2**) cells in the DRN that responded to 5-HT (**a**), 8-OH-DPAT (**b**), and DOI (**c**). The lower pie charts display the percentage of responses (inward *orange*, outward *green*, and no effect *white*) to 5-HT (**a**), 8-OH-DPAT (**b**), and DOI (**c**) in each type of cell. Scatter plots display the distributions of current amplitudes induced by 5-HT (**a3**), 8-OH-DPAT (**b3**), and DOI (**c3**) in the GFP(+) and mGFP(−) cells. The current amplitude was sorted by response (inward *orange*, outward *green*). *Boxes and whiskers* indicate mean ± standard error of the mean and the range from minimum to maximum, respectively

We next explored the pharmacological effects of 5-HT on the membrane current of DRN GFP(+) and mGFP(−) cells of GAD67^+/GFP^ mice. Prior studies have demonstrated that inhibitory effects are induced by 5-HT through the 5-HT_1A_ receptor in DRN 5-HT cells [6–8]. As shown previously, bath applications of 5-HT (10 μM) elicited outward currents in all tested mGFP(−) cells that were considered mostly 5-HT cells (*n* = 9, Fig. 3a2). However, in most tested GFP(+) cells, 5-HT elicited an inward current (77.8 %, *n* = 21/27; Fig. 3a1). 5-HT-induced outward currents were observed in a minority of GFP(+) cells (14.8 %, *n* = 4/27), and the remaining 7.4 % (2/27) of GFP(+) cells did not respond to 5-HT. These observations indicated that the 5-HT-mediated pharmacological properties of the membrane currents of DRN GFP(+) cells were heterogeneous.

Furthermore, we explored 5-HT-mediated responses in DRN GFP(+) and mGFP(−) cells. Because previous studies have demonstrated that the outward and inward currents elicited by 5-HT were mediated by the activation of 5-HT_1A_ and 5-HT_2A/2C_ receptors in DRN cells, respectively, we used selective agonists, such as 8-OH-DPAT and DOI for the 5-HT_1A_ and 5-HT_2A/2C_ receptor, respectively. Outward currents elicited by 8-OH-DPAT (5 μM) were observed in half of the GFP(+) cells (9/18) and 93 % of the mGFP(−) cells (14/15) (Fig. 3b1, b2). mGFP(−) cells had larger outward current amplitudes in response to 8-OH-DPAT than GFP(+) cells (GFP(+), 30.4 ± 4.0 pA vs. mGFP(−), 72.3 ± 8.3 pA, *p* < 0.001, *t* test; Fig. 3b3). In GFP(+) cells, the responses to 8-OH-DPAT varied [inward current, 16.7 % (*n* = 3/18); no response, 33.3 % (*n* = 6/18)]. These results suggested that DRN GFP(+) cells were heterogeneous for 5-HT receptor expression. We then examined whether the 5-HT–induced inward currents in GFP(+) cells involved 5HT_2A/2C_ receptor activation. As shown in Fig. 3c1, the membrane currents were recorded during the sequential application of 5-HT and DOI. Half of the tested GFP(+) cells (*n* = 7/14) showed a DOI-induced inward current, while 42.9 % (*n* = 6/14) of the GFP(+) cells had no response to DOI (Fig. 3c1). However, the DOI-induced inward current was detected in only a small population of tested mGFP(−) cells (18.2 %, 2/11; Fig. 3c2), and their amplitude was small or negligible (Fig. 3c3). Altogether, these data highlighted the heterogeneity of DRN GFP(+) cells in the regulatory effects through the activation of 5-HT receptors.

## Discussion

### Utility of GAD67^+/GFP^ mice

Present study is the first that characterized differences in electrophysiological and pharmacological properties of GABAergic and non-GABAergic cells in the DRN by using GAD67^+/GFP^ mice. To the best of our knowledge, there has been only 1 previous report about the electrophysiological properties of DRN GABAergic neurons [15]. Other studies that have used TPH immunoreactivity for cell typing have reported that the resting membrane potential, AP threshold, and input resistance did not statistically differ [6, 7]. This was probably because the tested non-5-HT neurons potentially contained heterogeneous cells and were not a uniform population. In this study, we demonstrated that GABAergic cells and 5-HT cells within the DRN were localized in distinct subregions (Fig. 1), which was similar to the findings of a previous report [3]. Furthermore, we showed that GFP-negative and TPH-immunonegative small cells were located in the lateral DRN, which was similar to the localization of GFP(+) cells. These cells were putatively glutamatergic because the neuronal cell types in the DRN, other than 5-HT cells, are mainly glutamatergic or GABAergic [2, 3, 16]. However, the properties of DRN glutamatergic cells remain to be elucidated.

Altogether, the identification of cell types with GAD67^+/GFP^ mice enabled a clearer discrimination of the properties of DRN cells.

### Intrinsic membrane properties of DRN GABAergic cells

The membrane properties of DRN GABAergic cells had distinctive features: more depolarized resting membrane potential, steeper AP threshold, and higher input resistance. These results suggested that GABAergic cells were easily excitable compared to 5-HT neurons. Moreover, the lower overshoot amplitude and fast AP kinetics may contribute to the high-frequency firing of up to ~25 Hz in the GABAergic cells. In fact, the input–output relationships induced by positive currents were linear until 100 pA of injection current, and they exhibited frequency adaptation over 100 pA (Fig. 2d).

### Physiological and pharmacological implications of DRN GABAergic cells

In the course of pharmacological experiments shown in Fig. 3, we could not exclude synaptic activity-driven indirect actions of serotonergic agonists. However, in most cases, spontaneous postsynaptic currents obtained from recorded cells were not altered by each agonist. Therefore, it is thought that the serotonergic agonist-induced membrane currents reflect direct action of the postsynaptic membrane.

It has been established that feedback inhibition through somatodendritic 5-HT_1A_ autoreceptors located on DRN 5-HT cells (Fig. 3a2, b2) is an important mechanism for controlling 5-HT neuronal activity [17, 18]. Like 5-HT cells, GABAergic cells play an important role in controlling 5-HT neuronal activity through 5-HT receptor-mediated modulatory actions [18, 19]. We found that 5-HT elicited excitatory responses in most GABAergic cells, while inhibitory actions were observed in 14.8 % of the GABAergic cells. However, these results cannot be explained simply by the activation of 5-HT_1A_ or 5-HT_2A/2C_ receptors in the subsequent experiments with 8-OH-DPAT or DOI. In the 8-OH-DPAT experiment, outward currents were observed in 50 % of the tested cells, which was a much higher cell population than that in the 5-HT challenge (14.8 %). However, it was comparable to immunohistochemical and in situ hybridization results that have reported that 40–50 and 10–15 %, respectively, of non5-HT neurons express the 5-HT_1A_ receptor [15, 20]. Therefore, the discrepancy between the 5-HT and 8-OH-DPAT results can be explained by the effects of other 5-HT receptor subtypes, such as the 5-HT_2A/2C_ receptor, which may overwhelm the response of 5-HT_1A_ receptor activation. Previous studies have shown that the balance between the 5-HT_1A_ and 5-HT_2A/2C_ receptors is important in the neuronal regulation by 5-HT [8, 21]. Moreover, the inward currents elicited by 8-OH-DPAT are probably due to the activation of the 5-HT_7_ receptor as 8-OH-DPAT has a moderate affinity for 5-HT_7_ receptors [22, 23].

For the selective 5-HT_2A/2C_ receptor-agonist experiment, DOI elicited inward currents in only 50 % of the tested GABAergic neurons (Fig. 3a3). Hence, only half of the 5-HT-induced inward currents were mediated by 5-HT_2A/2C_ receptor activation in GABAergic cells. The remaining half might be accounted for by the activation of the 5-HT_7_ receptor [18]. That is, in addition to the 5-HT_1A_ and 5-HT_2A/2C_ receptors, 5-HT_7_ receptors might be implicated in the postsynaptic effects of DRN GABAergic neurons. Actually, the 5-HT_7_ receptor, which is expressed in some GABAergic neurons, contributes to GABA release to regulated 5-HT neurons in the DRN [24, 25].

In heterogeneous DRN neurons, competitive/synergistic actions of multiple 5-HT receptor subtypes induce distinct signaling properties and contribute to a diverse 5-HT neurotransmitter system. These complex regulations have been implicated in the etiology and treatment of many common psychiatric disorders. More extensive explorations of 5-HT receptor subtypes, including their properties, distributions, and interactions, are necessary for the development of novel therapeutic strategies that target 5-HT receptor subtypes.
